# Unveil the transcriptional landscape at the *Cryptococcus*-host axis in mice and nonhuman primates

**DOI:** 10.1371/journal.pntd.0007566

**Published:** 2019-07-22

**Authors:** Hailong Li, Yanjian Li, Tianshu Sun, Wei Du, Chao Li, Chenhao Suo, Yang Meng, Qiaojing Liang, Tian Lan, Manli Zhong, Sheng Yang, Cheng Niu, Dancheng Li, Chen Ding

**Affiliations:** 1 College of Life and Health Sciences, Northeastern University, Shenyang, Liaoning, China; 2 Beijing Key Laboratory for Mechanisms Research and Precision Diagnosis of Invasive Fungal Diseases, Beijing, China; 3 Department of Scientific Research, Central Laboratory, Peking Union Medical College Hospital, Chinese Academy of Medical Science, Beijing, China; 4 Software College, Northeastern University, Liaoning, Shenyang, China; 5 Key Laboratory of Data Analytics and Optimization for Smart Industry, Northeastern University, Shenyang, Liaoning, China; Rutgers University, UNITED STATES

## Abstract

Pathogens and hosts require rapid modulation of virulence and defense mechanisms at the infection axis, but monitoring such modulations is challenging. In studying the human fungal pathogen *Cryptococcus neoformans*, mouse and rabbit infection models are often employed to shed light on the disease mechanisms but that may not be clinically relevant. In this study, we developed an animal infection model using the non-human primate cynomolgus monkey *Macaca fascicularis*. In addition, we systematically profiled and compared transcriptional responses between the infected mice and the cynomolgus monkey, using simultaneous or dual RNA next-generation sequencing. We demonstrated that there are shared but distinct transcriptional profiles between the two models following *C*. *neoformans* infection. Specifically, genes involved in immune and inflammatory responses are all upregulated. Osteoclastogenesis and insulin signaling are also significantly co-regulated in both models and disrupting an osteoclastogenesis-associated gene (OC-STAMP) or the insulin-signaling process significantly altered the host tolerance to *C*. *neoformans*. Moreover, *C*. *neoformans* was shown to activate metal sequestration, dampen the sugar metabolism, and control cell morphology during infection. Taking together, we described the development of a non-human primate model of cryptococcosis that allowed us to perform an in-depth analysis and comparison of transcriptome profiles during infections of two animal models and conceptually identify host genes important in disease responses. This study provides new insights in understanding fungal pathogenesis mechanisms that potentially facilitate the identification of novel drug targets for the treatment of cryptococcal infection.

## Author summary

The host-pathogen interaction is highly dynamic and tightly regulated, and yet is difficult to monitor. Traditional investigations provide valuable information for the understanding of pathogenic microbial biology but are time-consuming and often neglect the host immune responses. In addition, current animal models for studying pathogenic fungi are limited in mimicking the responses from humans. The development of a new *Cryptococcus neoformans* infection model using nonhuman primates and the utilization of simultaneous RNA sequencing analysis provide fast and clinically relevant research data allowing the identification of novel critical players from both the invading fungus and the host. The data from the current study would not only help to decipher disease mechanisms but also promote the discovery of novel antifungal drug targets.

## Introduction

Microbial pathogens including fungi have evolved rapid and precise yet complex gene expression regulatory mechanisms to colonize hosts during infections [1–3]. To counter pathogenic invasions, the host cells often trigger a series of response cascades, by restricting essential nutrients, producing cytokines and chemokines, or inducing immune cell infiltration that activates defense mechanisms [4, 5]. Monitoring transcription alterations and characterizing gene functions at the pathogen-host axis is critical for understanding infectious disease mechanisms and managing treatment strategies.

*Cryptococcus neoformans* is a basidiomycete fungal pathogen that infects primarily immunodeficient individuals causing meningoencephalitis. In studying cryptococcal pathogenesis mechanisms, forward genetics and murine infection models are often employed to assess factors involved in infection. Such efforts have resulted in the identification of virulence-modulating genes involved in capsule and melanin production, morphological switching, metal homeostasis, and others [6–11]. However, fungal virulence is often a multifaceted trait and additional factors contributed to virulence are present. Based on the *C*. *neoformans* genome databases (http://fungidb.org/fungidb/ and https://www.ncbi.nlm.nih.gov), nearly half the genes encode hypothetical proteins and their expression and functions remain unknown. Traditional investigations in *C*. *neoformans* often employ *in vitro* and *in vivo* models that serve as sensors or reporters of the “host” responses. For instance, fungal insertion gene disruption plus a murine infection model have revealed that fungal iron and copper homeostasis play reciprocal functions at various host niches [9, 12, 13]. Such studies indicated that host cells might differentially manipulate metals during fungal infection but further investigation is limited by the approach. Additional limitations also include that the mouse model may not be clinically relevant.

Assessing the transcriptomes of microbe and host at the infection axis would shed new insights in understanding the disease mechanisms but the approach is challenging due to the dynamic nature of infection and specific but complex natures of the events. The newly developed dual or simultaneous RNA-sequencing (RNA-Seq) technique allows a rapid and global capture of the transcriptome profiles of invading microbe and the host [14–16]. This has been extensively applied in dissecting the pathogenicity of bacterial pathogens and transcriptome responses of infected cell lines and mouse tissues [17]. Another important concern with the current fungal infection studies are due to evolutionary divergence between the rodents and humans reflected by drastic differences in immune responses.

To overcome the lack of an efficient approach evaluating gene expressions of both the microbe and the host and the limitation of clinical relevance of the mouse model in studying *C*. *neoformans* pathogenesis, we successfully developed a *C*. *neoformans* pulmonary infection model using the cynomolgus monkey *Macaca fascicularis*. Dual RNA-Seq was performed and results were compared. We found that less than 20% (225) of the differentially expressed genes were shared between two models, indicating distinct species-specific responses. We also found that genes involved in diabetes and osteoclastogenesis were significantly altered in response to *C*. *neoformans* infection. Moreover, a comparison between mouse and monkey lung tissue expression profiles revealed other processes important in infection of both species. Collectively, we have successfully developed a pulmonary cryptococcosis model of nonhuman primates that allowed dissection of factors important in interactions between *C*. *neoformans* and model organisms.

## Results

### Pulmonary cryptococcal model using *M*. *fascicularis* and dual RNA-Seq

**Fig 1:**
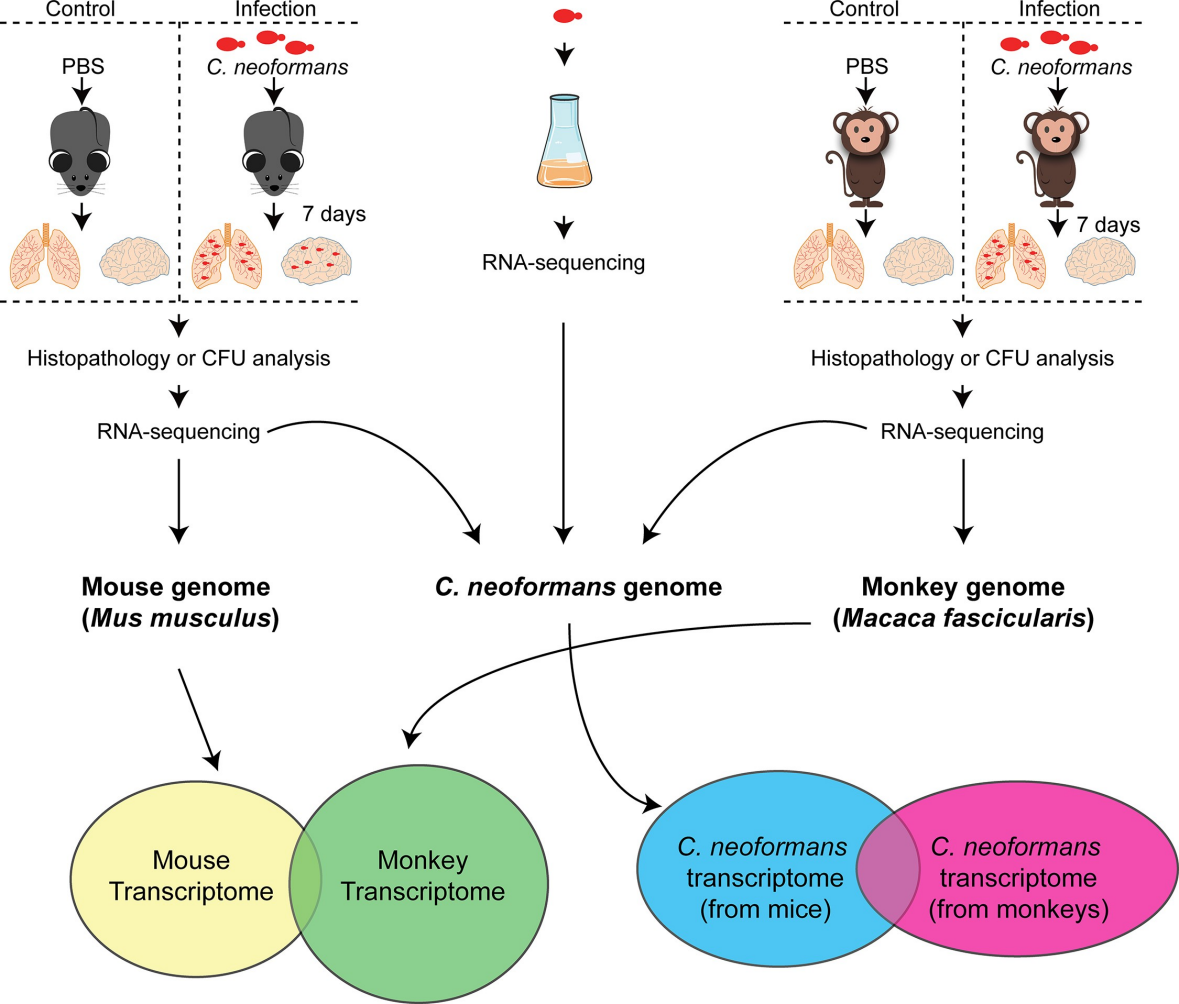
Experimental flow chart. The wild type *C*. *neoformans* strain H99 was grown in YPD medium. Cells were then washed and counted, and divided into three groups: YPD control group, mouse infection group, and monkey infection group. For the control group, fungal cells were grown in YPD medium at 37°C and 200 rpm for 12 hours, washed, and maintained in RNAlater at -80°C. Animals were infected via intratracheal injection in three C57BL/6 female mice and three female *Macaca fascicularis*. Three mice and three monkeys were also infected with PBS buffer as controls. Animal tissues were collected and processed for either library construction, histology observation, or CFU determination.

**Fig 2:**
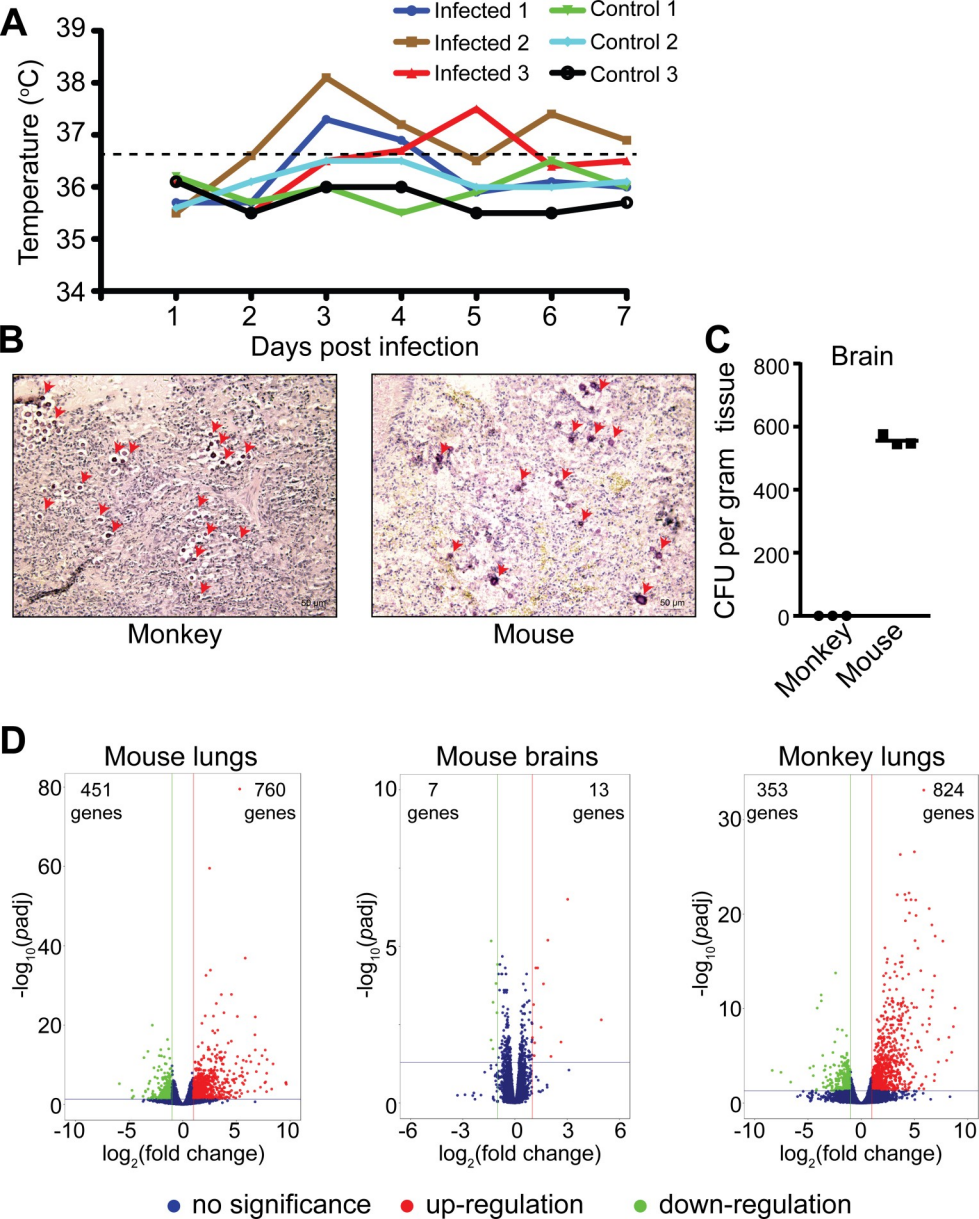
RNA-Seq of mice and monkeys infected with *C*. *neoformans*. (A) Back temperature was used to indicate body temperature in monkeys and that was measured every 24 hours following infection. (B) Histopathology studies of infected mouse and monkey lung tissues fixed and sectioned at 10 μm thickness, then stained with mucicarmine. *Cryptococcus neoformans* cells are indicated by red arrows (scale bar = 50 μm). (C)*Cryptococcus neoformans* colony forming units (CFUs) in mouse and monkey brain tissues. Infected brain samples were homogenized and plated onto YPD agar plates. Five random monkey brain samples were pooled to maximize detection of fungal cells. The YPD agar plates were incubated at 30°C for 3 days. Fungal cell colonies were counted, and CFUs were calculated. (D) Volcano plots of detected transcripts. Normalized transcripts from infected mouse lung and brain tissues and infected monkey lung tissues are plotted. When transcript changes are at least 2-fold with *p-adj* < 0.05, the genes were considered significantly differentially expressed. Red dots indicate induced transcripts, and green dots indicate repressed transcripts. The numbers of differentially expressed transcripts are indicated.

A flowchart of animal studies is illustrated in Fig 1. Considering the limitations and difficulties in accessing *C*. *neoformans-*infected human materials, a nonhuman primate pulmonary infection model was developed using the cynomolgus monkey *M*. *fascicularis*. The monkeys were infected intratracheally with 1x10^4^ cells/kg body weight of *C*. *neoformans* strain H99. Following the infection, clinical signs for pulmonary disease developed in monkeys within 48 hours. These signs or symptoms included cough, reduced appetite and movement, and increased body temperature. An increasing in body temperature following the infection indicated an acute response to infection (Fig 2A) that was substantiated by lung tissue staining with mucicarmine that showed the presence of *C*. *neoformans* capsular structure within the lungs (Fig 2B). This is similar to H99 strain infection of C57BL/6 mice, done simultaneously as a comparison (Fig 2B). At 7 days postinfection, *C*. *neoformans* cells were detected in the brain of mice, but not of monkeys (Fig 2C), which may be due to the monkeys were not immune suppressed or demonstrate a sign of clearance of infection. For this reason, monkey RNA-Seq involved only on the monkey lung tissue, whereas mouse RNA-Seq used both brain and lung tissues.

High-throughput RNA-Seq was performed and data reproducibility was confirmed (S1 Fig). Expression of 18,935 transcripts in *C*. *neoformans* infected mice were detected. Changes of at least 2-fold, either up or down, and the adjusted *p* value (*p-adj*) of 0.05 was applied. Of all of the transcripts detected in the mouse lungs, 760 genes were significantly increased while 451 genes were decreased. In the infected monkeys, 824 genes were significantly increased whereas 353 genes were decreased. (Fig 2D and S1 Table). Despite a significant amount of *C*. *neoformans* cells were detected in the brain, only 20 transcripts were found to be differentially expressed in the mouse brain and among them 13 were up-regulated and 7 were down-regulated (Fig 2D and S1 Table). This is most likely due to the brain tissue considered as the “immune privilege”, where *C*. *neoformans* has been shown to persist as “latent” infections [9, 18, 19].

### Mouse and monkey immune responses to *C*. *neoformans* infection

**Fig 3:**
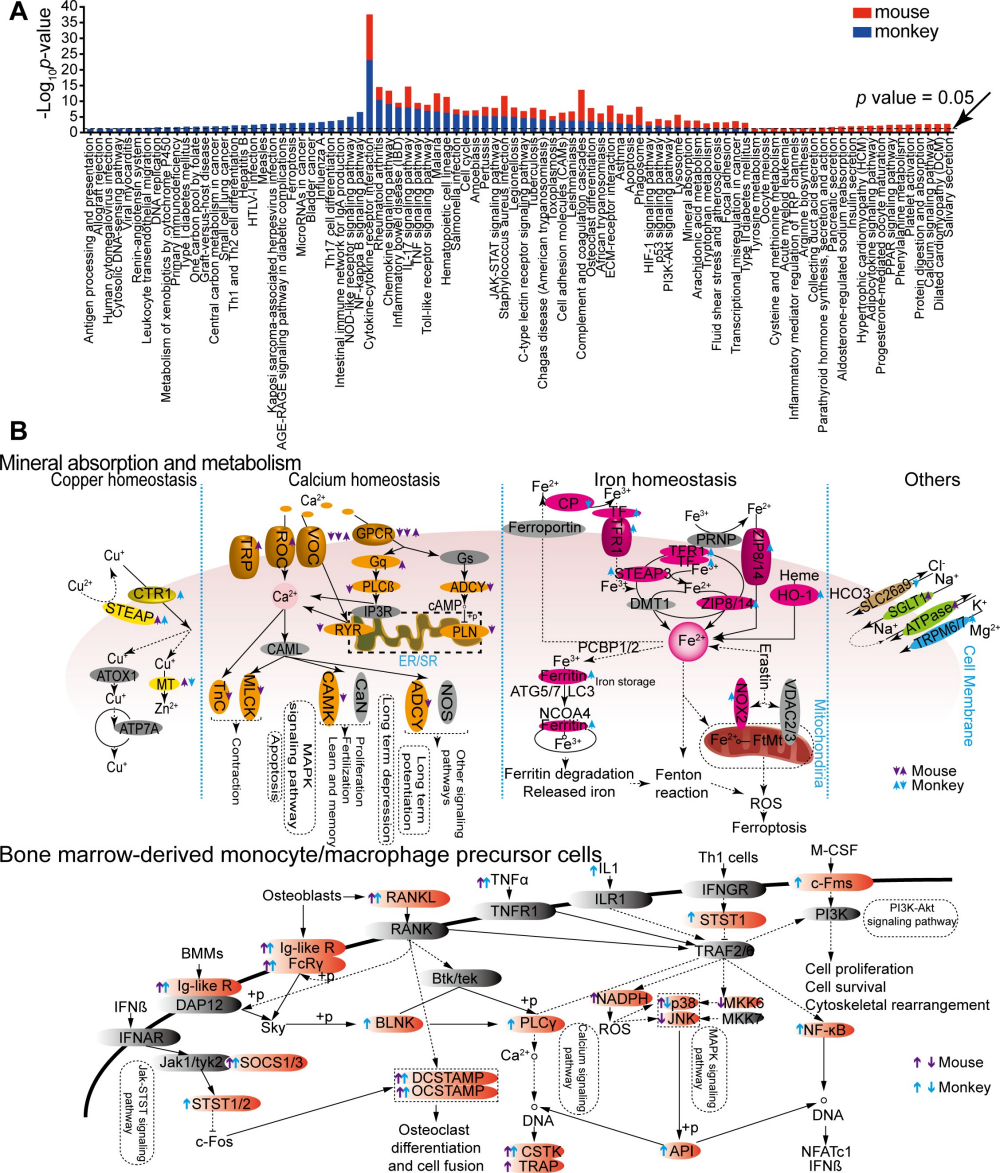
KEGG analyses of *C*. *neoformans*-infected lung tissues from mice and monkeys. (A) Significantly expressed transcripts were subjected to KEGG analyses using Clusterprofiler 3.6.0 and plotted. Values in blue are for monkey lungs and values in red are for mice lung tissues. The dashed line separates values that meet the significance test (*p* < 0.05) from those that do not. (B) Differentially expressed metal and bone homeostasis genes (arrows). Upward arrows indicate induction of gene expression, and downward arrows indicate repression of gene expression. Monkey genes are indicated by blue arrows, and mouse genes are indicated by purple arrows.

To categorize differentially expressed transcripts and characterize their functions, KEGG (Kyoto Encyclopedia of Genes and Genomes) analyses were employed. In total, 40 KEGG pathways were significantly enriched in both data sets (Fig 3A and S2 Table). Pathways such as the cytokine-cytokine receptor interaction, the chemokine signaling pathway, the TNF (tumor necrosis factor) signaling pathway, and the toll-like receptor signaling pathway were all drastically induced during pulmonary infection of mice and monkeys (Fig 3A and S2 Table). Notably, the host transcriptome in response to *C*. *neoformans* infections resembled that of other infectious or inflammatory diseases including rheumatoid arthritis, malaria infection, salmonella infection, tuberculosis infection, asthma, and diabetes. The IL-17 signaling pathway was also increased in response to *C*. *neoformans* infection. The occurrence of IL-17-producing CD4+ cells and IL-17 were shown to have a critical function against fungal proliferation in the host [20–22].

Fifty-nine gene transcripts of genes encoding cytokines and chemokines were differentially expressed in infected mouse lung tissues, in contrast to 78 found in monkey tissues (S2 Fig). While 35 inflammatory-associated gene transcripts were increased in both mice and monkeys, genes encoding the transcription factor VEG1 was differentially regulated between two models (down-regulated in mice and up-regulated in monkeys) (S2 Fig). Twenty chemokine transcripts (including CC and CXC subfamilies) were significantly induced in the lung tissues of both species (e.g., CCL1, CCL2, CCL3, CCL4, CXCL1, CXCL5, CXCL6, and CXCL17) (S2 Fig). While the functions of many induced cytokines in *C*. *neoformans* infection remain not known, clinical investigations have demonstrated that CCL2 and CCL3 are induced in meningitis-forming patients [23, 24]. Moreover, CXCL10, which was specifically induced in monkey lung tissues, was shown to be abundantly expressed in patients with cryptococcosis[24]. This data indicated that the *C*. *neoformans* monkey infection model could recapitulate the immune responses of humans.

IL2RA, CSF2RB and CSF2RA chemokine receptor genes were highly expressed during *C*. *neoformans* pulmonary infection in both mice and monkeys. The transcripts of TNF and RANKL (the TNF family) and of GDF15 and INHBA (the TGF-ß family) were also induced in mice and monkeys upon infection by *C*. *neoformans* (S2 Fig). Nevertheless, species-specific transcriptome variations were also found. For example, IL1A and IL1B gene expression were induced in monkey lungs, but not in mice. Instead, there was the evidence that IL1RN, an IL1 receptor antagonist, was induced (S2 Fig). Collectively, the results revealed a global immunological response to pulmonary infection by *C*. *neoformans* in both murine and nonhuman primate models. Importantly, the monkey inflammatory results resemble those from clinical infection of humans.

### Host metal homeostasis following *C*. *neoformans* infection

Previous studies have demonstrated that metal homeostasis plays an important role during fungal colonization of host tissues[9, 12, 13]. Consistent with these studies, our results showed alterations in gene expression specific to metal homeostasis in cells of either *C*. *neoformans* or the host. The increased expression of the cupric reductase gene, *STEAP*, indicated that Cu^2+^ could be converted to Cu^+^ by the host cells and is being transported via the high-affinity mammalian copper transporter, CTR1. The gene expression of *CTR1* is induced in monkey tissues, but *CTR1* transcripts were not induced in mice even though other studies have demonstrated that *C*. *neoformans* induces mouse CTR1 protein species in alveolar immune cells (Fig 3B) [13]. We speculate that CTR1 regulation by *C*. *neoformans* occurs through gene expression modulation in monkeys, but via posttranscriptional regulation in mice.

Several genes involved in iron acquisition (*TFR1* and *ZIP8/14*), iron transportation (transferrin), and iron storage (ferritin) were increased in the infected monkey lung tissues. Interestingly, *NOX2*, a reactive oxygen species-generating enzyme that causes ferroptosis, was also highly induced in monkeys. However, this was not the case for the mouse as most of these genes remained unchanged (Fig 3B). In contrast, the genes involved in calcium homeostasis are largely repressed in mice which remained constant in monkeys. These data suggested that, despite the shared common response in copper homeostasis, mouse and monkey differed in iron and calcium homeostasis following *C*. *neoformans* infection (Fig 3B).

### *C*. *neoformans* infection perturbs osteoclastogenesis signaling

Dual RNA-Seq also revealed significant changes in the gene expression of osteoblast-related genes. The expression of the receptor activator of the nuclear factor kappa-B ligand (RANKL), whose expression is critical for bone regeneration and remodeling [25], was induced in both animal models. The gene-encoding osteoclast stimulatory transmembrane protein (OC-STAMP), which is induced by RANKL and involved in the promotion of osteoblast differentiation and macrophage cell-cell fusion [26], is also significantly induced (Fig 3C). Therefore, due to the important function of CO-STAMP in modulating macrophages that are the first line of defense against *C*. *neoformans* infection, our results hinted that these osteoblast differentiation regulators might have important functions during *C*. *neoformans* pulmonary infections.

### Shared anti-fungal mechanisms between mice and monkeys

**Fig 4:**
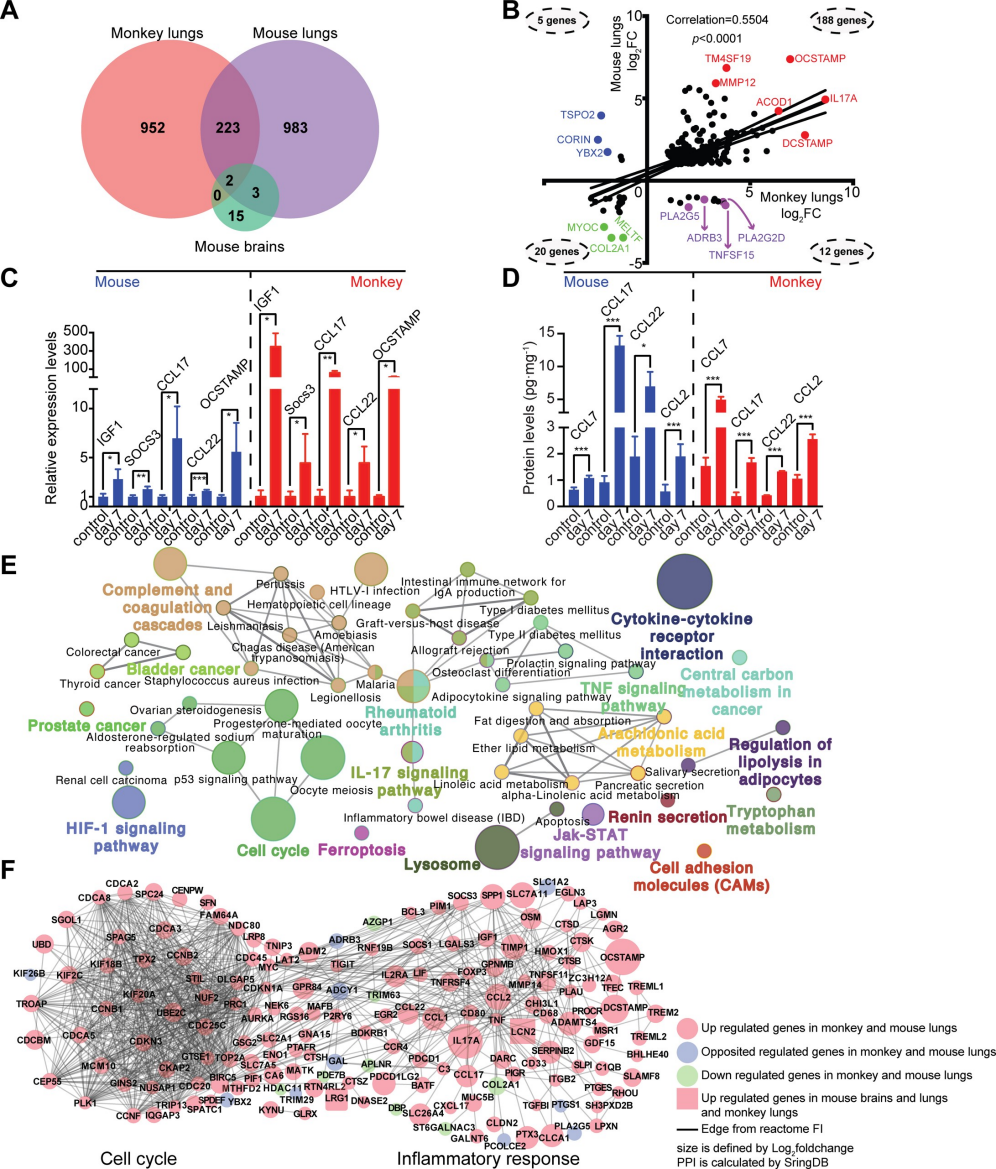
Correlations between *C*. *neoformans*-infected mouse and monkey tissues. (A) Differentially expressed mouse and monkey genes analyzed using a Venn diagram. (B) Correlations and *p* values for gene expression patterns of 225 overlapping genes. The number of genes in each group are indicated. (C) RT-qPCR assays were performed for IGF1, SOCS3, CCL17, CCL22 and OC-STAMP. Three biological replicates were performed for each gene. *Student t test* was performed. *p < 0.05, ** *p* < 0.01, *or* ***p < 0.005. (D) The production of chemokines was quantified using ELISA. Three biological replicates were performed for each gene. *Student t test* was performed. *p < 0.05, ** *p* < 0.01, *or* ***p < 0.005. (E) The gene ontology (GO) regulation network of 225 genes. After identifying the human orthologs of 225 genes using Biomart (http://www.biomart.org), the enriched GO terms were calculated using Cytoscape 3.6.0 and ClueGO v2.5.2 employing medium network specificity. (F) Regulation network of overlapping genes. The regulation relationships between 225 overlapping genes were calculated using StringDB version 10.5 (https://string-db.org). Of those, 167 gene products (nodes) were mapped, and 976 relationships (edges) were located in the network. The network was constructed using Cytoscape. Pink circles indicate induced genes in monkey and mouse lungs. Blue circles indicate genes that are reciprocally expressed between mice and monkeys. Green circles indicate repressed genes in monkey and mouse lungs. Pink squares indicate genes that are up-regulated under all conditions.

The transcripts of 225 genes showed significant changes in expression in infected mouse and monkey lung tissues, representing 19.1% of the differentially expressed transcripts in monkeys and 18.6% of those in mice (Fig 4A). The regulation patterns of 225 genes between mouse and monkey lung transcripts were analyzed (*p* < 0.001 and correlation (*R*) = 0.5504) (Fig 4B). Overall, 208 genes (188 up-regulated and 20 down-regulated) demonstrated similar regulation patterns in the lungs of both mice and monkeys, while 17 showed differential regulations. Of 188 co-up-regulated genes in mice and monkeys, *MMP12*, *IL17A*, and *OC-STAMP* were the most increased in infected lungs (Fig 4B). Random targets were selected for real-time PCR and ELISA validations that were consistent with the transcriptome results (Fig 4C and Fig 4D).

The 255 shared genes were then used for gene ontology (GO) analysis for regulatory network constructions. These genes were shown to be significantly enriched in the cell cycle regulation, lysosomes, cytokine-cytokine receptor interactions, ferroptosis, the hypoxia response, and the IL17 signaling pathway (Fig 4E). A gene product regulation network was constructed based on human reactome information. Together, 167 gene products (nodes) were mapped, and 976 regulations (edges) were identified in the network (Fig 4F). All nodes were clustered into two groups: the cell cycle and inflammatory responses. Genes involved in cell growth and proliferation such as *CCNB1*, *CCNF*, *CDC20*, and *MYC*, were identified, strongly demonstrating that *C*. *neoformans* infection promotes differentiation of pulmonary immune cells.

### OC-STAMP is a negative regulator during pulmonary *C*. *neoformans* infection

Our results showed the dramatic inductions in the expressions of genes important in immune cell responses of both mice and monkeys, including *OC-STAMP* and matrix metallopeptidase 12 [*MMP12*] (S1 Table and Fig 4B). Since the increase of *MMP12* mRNA levels in response to *C*. *neoformans* infection has been demonstrated previously [27], we sought to further explore the potential function *of OC-STAMP* and *MMP12* in pulmonary infection by *C*. *neoformans*.

**Fig 5:**
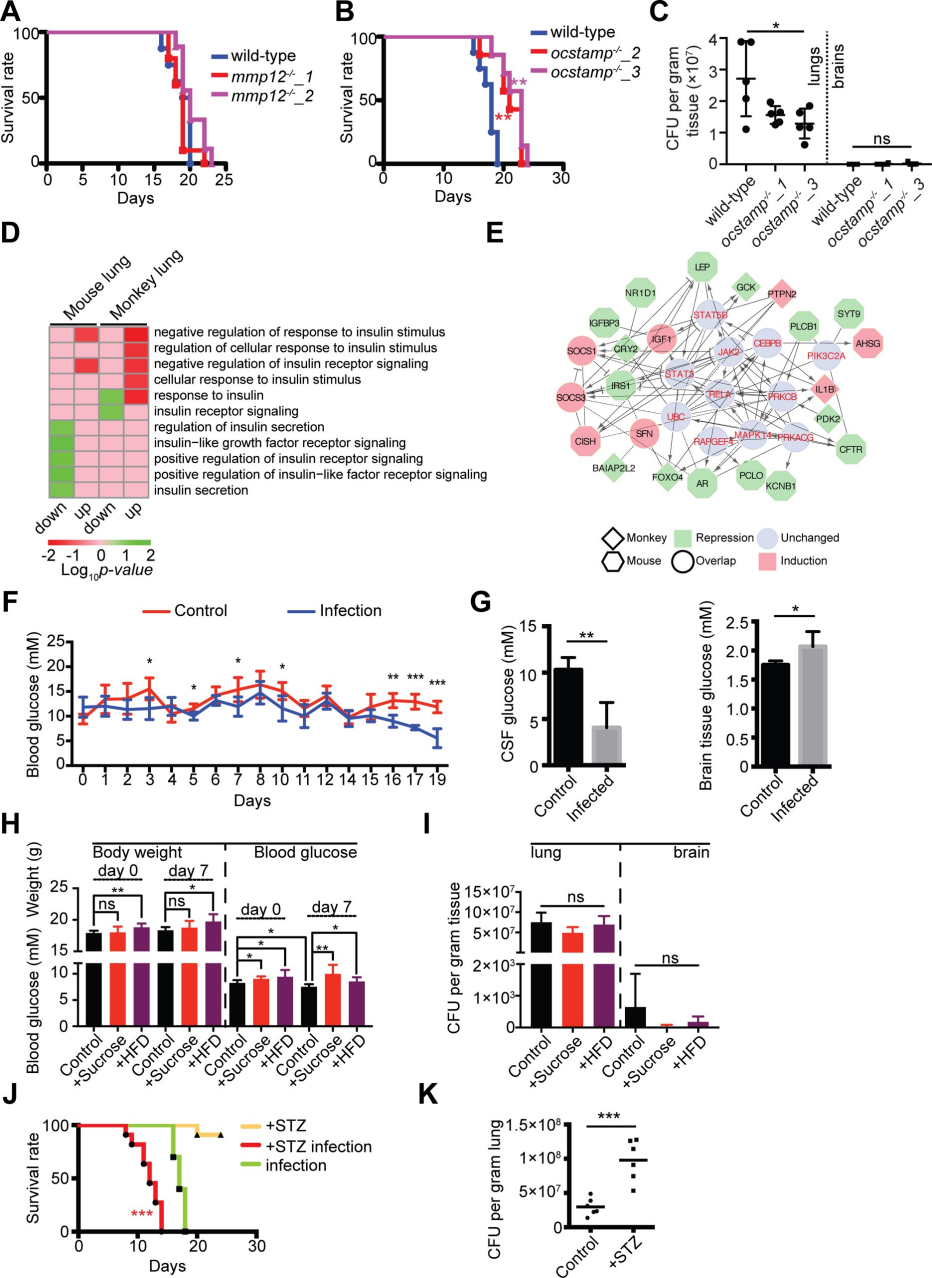
Host OC-STAMP and insulin-signaling play important roles in *Cryptococcus neoformans* infection. (A) Survival curve of *C*. *neoformans*-infected *MMP12*^*-/-*^ mice. *Cryptococcus neoformans* strain H99 was used to infect wild-type C57BL/6 mice (*n* = 10) and two lines of *MMP12*^*-/-*^ female mice (*MMP12*^*-/-*^_1 mice, *n* = 7; *MMP12*^*-/-*^_2 mice, *n* = 8). The intranasal route of infection was employed, and animals were monitored daily for signs of infection. Statistical analysis was performed using the log-rank test. (B) Survival curve of *C*. *neoformans-*infected *OC-STAMP12*^*-/-*^ mice. The H99 strain was used to infect the wild-type C57BL/6 mice (*n* = 8), and two lines of *OC-STAMP*^*-/-*^ female mice (*OC-STAMP*^*-/-*^_2 mice, *n* = 8; *OC-STAMP* ^*-/-*^_3 mice, *n* = 7) via the intranasal route of infection was applied, and animals were monitored for signs of infection. Statistical analysis was performed using the log-rank test (**p < 0.01). (C) Mouse lung tissues of *C*. *neoformans*-infected *OC-STAMP* ^*-/-*^ mice were assayed for CFUs. Infections were performed as described in Fig 5C (*n* = 5). Lung and brain tissues were removed 14 days postinfection, then homogenized and plated onto YPD agar plates. Fungal cells were counted, and CFUs were calculated. Statistical analysis was performed using one-way ANOVA (*p < 0.05). (D) Gene ontology for insulin-associated biological processes was calculated. Terms related to insulin action are shown. (E) Gene regulation network for genes significantly enriched based on GO (Fig 5D). Human orthologs were identified, and the reactome network was constructed using the human reactome database. (F) Blood glucose tests in *C*. *neoformans* infected mice. Ten C57BL/6 mice were intranasally inoculated with the H99 strain. Ten additional mice were used as controls, sham infected with PBS buffer. Glucose levels were measured using blood samples from tail veins. The Student *t* test was performed (**p < 0.01, ***p < 0.005). (G) Glucose alterations in the cerebrospinal fluid (CSF) and brain tissues from infected mice. Controls (*n* = 4) and infected mice (*n* = 4) were humanely killed 19 days postinfection, and CSF samples were isolated. Brain tissues were homogenized. Glucose levels were measured. The Student *t* test was performed (**p < 0.01, *p < 0.05). (H) Mice were treated with high sugar- or fat-diet. Mice were divided into three groups (n = 7 each group): control group (normal chow and water), high sugar group (normal chow and 30% w/v sucrose), and high fat group (high fat-diet, HFD and normal water). Body weights and blood sugar levels were measured. (I) CFU analysis of high sugar- and fat-diet treated mice. Mice from Fig 5H was used for CFU assays. ns indicates no significant change. (J) Infection survival assays in insulin-deficient mice. C57BL/6 female mice were divided into three groups: the STZ group, STZ infection group, and infection group (*n* = 10 each). In the first two, STZ (150 mg/kg) was injected, and blood glucose levels were monitored. When the glucose level was greater than 300 mg/dL the mouse was used for further analysis. The two infection groups were infected with the H99 strain of *C*. *neoformans* via the intranasal route. Animals were monitored for sickness and signs of infection. Statistical analysis was performed using the log-rank test (***p < 0.005). (K) The CFU analysis of STZ-treated mice. The infection experiment was performed as described in Fig 5H (*n* = 6). Animals were humanely killed 11 days postinfection, and CFUs from lung tissues were counted and calculated. The Student *t* test was performed (*** *p* < 0.005).

The CRISPR-CAS9 gene editing system was employed to generate knockout mice in which the expression of *MMP12* and *OC-STAMP* was systematically depleted (S3A Fig). Two knockout lines, each harboring a codon-shift mutation in the for *MMP12* gene and three knockout lines of *OC-STAMP* were generated (S3B and S3C Fig). Further virulence testing revealed that MMP12 has no obvious function in defending *C*. *neoformans* (Fig 5A), while the *OC-STAMP*^*-/-*^ knockout mice showed improved survival by 5 days (Fig 5B). This finding was consistent with the lung fungal burden assay (Fig 5C). Fungal cells were significantly decreased in the lungs of knockout mice, but the CFUs remained unchanged in the brain. While further validation remains needed, the data suggested that OC-STAMP plays a role against *C*. *neoformans* during pulmonary infection.

### Insulin signaling process is modulated during *C*. *neoformans* infection

GO analysis revealed significantly enriched GO terms for responses to insulin stimuli in the lungs and insulin and insulin receptors were significantly enriched and induced in both mice and monkeys (Fig 5D). Insulin receptor signaling was down-regulated in monkeys and insulin secretion and insulin receptor signaling were repressed in mice. Twenty-six genes corresponding to the GO terms were differentially expressed. Out of these 26, 25 (except *MZB1*) were mapped using reactome analysis (Fig 5E). Specific to the monkeys, nine gene expressions were altered: 6 down-regulated (*GCK*, *FOXO4*, *CAV2*, *CRY2*, *PDK2*, and *BAIAP2L2*) and 3 up-regulated (*CISH*, *PTPN2*, and *IL1-β*). Specific to mice, twelve gene expressions were altered: 11 down-regulated (*LEP*, *PLCB1*, *SYT9*, *IGFBP3*, *CACNA1E*, *PCLO*, *NR1D1*, *AR*, *IRS1*, *KCNB1*, and *CFTR*) and 1 up-regulated (*AHSG*). Moreover, reactome analysis implicated 11 genes in network regulation; however, they were unchanged in our RNA-Seq analysis. Four insulin-associated genes were similarly induced in mice and monkeys (*SOCS1*, *SOCS3*, *IGF1*, and *SFN*). *SOCS1* and *SOCS3* are thought to promote the ubiquitin-mediated degradation of *IRS1* and *IRS2* that are important signaling proteins in insulin function [28], whereas *SFN* provides a reciprocal function to insulin in regulating the expression of collagenase mRNA in dermal fibroblasts [29].

These results strongly suggested that the host insulin signaling has a critical role during pulmonary infection by *C*. *neoformans* infection. Indeed, diabetic patients have been shown to be more susceptible to pulmonary infections such as tuberculosis [30], but the correlation between insulin signal and *C*. *neoformans* infection has not yet been investigated. Therefore, we first address whether *C*. *neoformans* infection could modulate glucose levels in the host by monitoring daily blood glucose levels for 19 days following the infection. Indeed, glucose levels of infected mice remained low in comparison to the control animals. Significant reduced blood sugar levels were detected at 3-day, 5-day, 7-day, 10-day and drastically dropped 16-day post infection (Fig 5F). We also measured glucose levels in the lung, brain, and cerebrospinal fluid (Fig 5G). While no changes were detected in the lungs due to detection limitation, a decrease was found in the cerebrospinal fluid while an increase was found in the brain. These alterations occurred in a late stage of infection. Next, the host sugar status was disturbed by supplementing high sugar- and fat-diet. However, modulating the host sugar levels has no impact on the tolerance to *C*. *neoformans* infection (Fig 5H and Fig 5I).

Lastly, we utilized streptozotocin (STZ) to induce diabetes in mice prior to *C*. *neoformans* infection. We found that STZ-treated mice were more susceptible to *C*. *neoformans* infections based on survival rates and fungal burdens (Fig 5J). All mice reached the endpoint by the 14th day postinfection with a profound fungal burden in the lungs (Fig 5K). These results suggested that diabetic individuals have a significant higher susceptibility to *C*. *neoformans* infection.

### Dual RNA-Seq reveals *C*. *neoformans* responses specific to mice and monkeys

**Fig 6:**
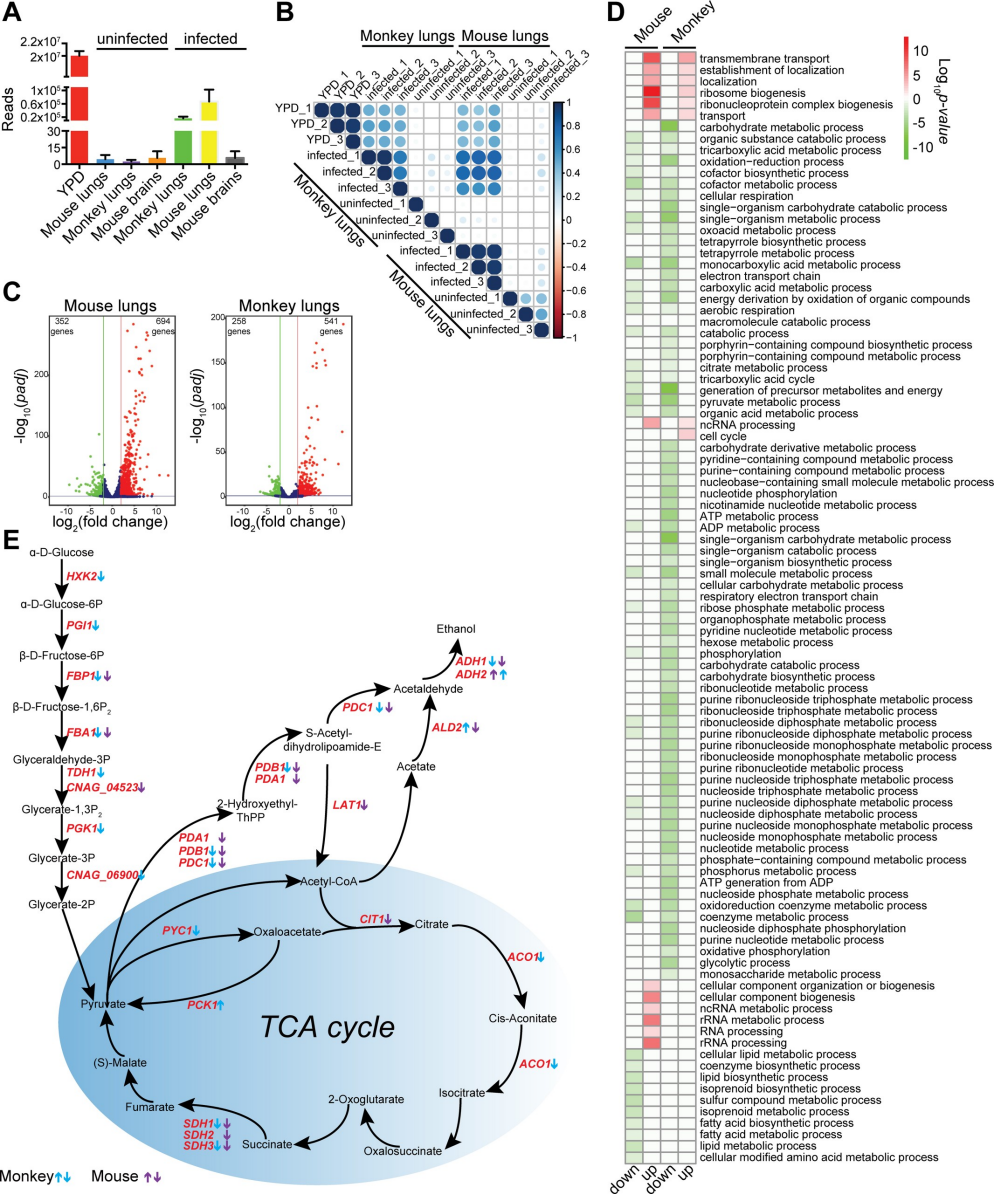
*Cryptococcus neoformans* transcriptional profiles from lung tissues of mice and monkeys. (A)Read counts of *C*. *neoformans* dual RNA sequences mapped to the *C*. *neoformans* genome. Genome-matched reads were counted and plotted, showing an average of three replicates. Standard deviations are as shown. (B) Correlations between *C*. *neoformans* dual RNA data. *Cryptococcus neoformans* genome-matched reads were compared, and correlations were determined using the corrplot package version 0.84. (C) Volcano plots of normalized *C*. *neoformans* transcripts from infected mouse lung and monkey lung tissues. Transcripts induced or repressed at least 4-fold with *p-adj* < 0.05 were considered significantly differentially expressed. Red dots represent induced transcripts, and green dots indicate repressed transcripts. The numbers of differentially expressed transcripts are indicated. (D)Gene ontology analysis of *C*. *neoformans* genes from mouse and monkey lung tissues. Significantly enriched GO terms are shown. (E) Gene regulation of glycolysis and the TCA cycle during pulmonary *C*. *neoformans* infection of mice and monkeys. Gene regulation patterns are indicated by arrows, indicating mouse genes in purple and monkey genes in blue.

To gain knowledge about how *C*. *neoformans* survives in the host, the transcriptome data was compared with that from cells grown in liquid YPD medium. Overall, 6920 transcripts were found out of 60,041,390 mapped read-counts (YPD) media, 6445 transcripts out of 200,724 mapped read-counts (mice), and 5671 transcripts out of 48,406 mapped read-counts (monkeys) (Fig 6A). Reads from uninfected lung and brain (mice) and lung (monkeys) were mapped to the *C*. *neoformans* genome as controls, which resulted in an extremely low number of reads, strongly implicating that our data was of high quality.

Correlation tests were performed for all data sets, and strong correlations were found between mice and monkeys. Good correlations were found for transcriptomes both *in vivo* (mice and monkeys) and *in vitro* (YPD) (Fig 6B). In the mouse infection model, 694 genes were up-regulated at least 4-fold versus cells grown *in vitro*, whereas 352 genes were significantly down-regulated at least 4-fold. In the infected lungs of monkeys, 541 genes were significantly induced in gene expression, while 258 genes were repressed (S3 Table and Fig 6C).

GO analysis was performed that revealed that, despite that common biological processes are used when invading these two hosts, *C*. *neoformans* cells behaved differently in rodents than in nonhuman primates. The common theme is that *C*. *neoformans* induces ribosome biogenesis, transmembrane transport, and ncRNA processing and down-regulating cellular respiration, and phosphorylation (Fig 6D, S4 and S5 Tables). In particular, genes involved in the glycolytic process and the TCA cycle was down-regulated (Fig 6E, S4 and S5 Tables).

### Identifying novel factors contributing to the virulence of *C*. *neoformans*

**Fig 7:**
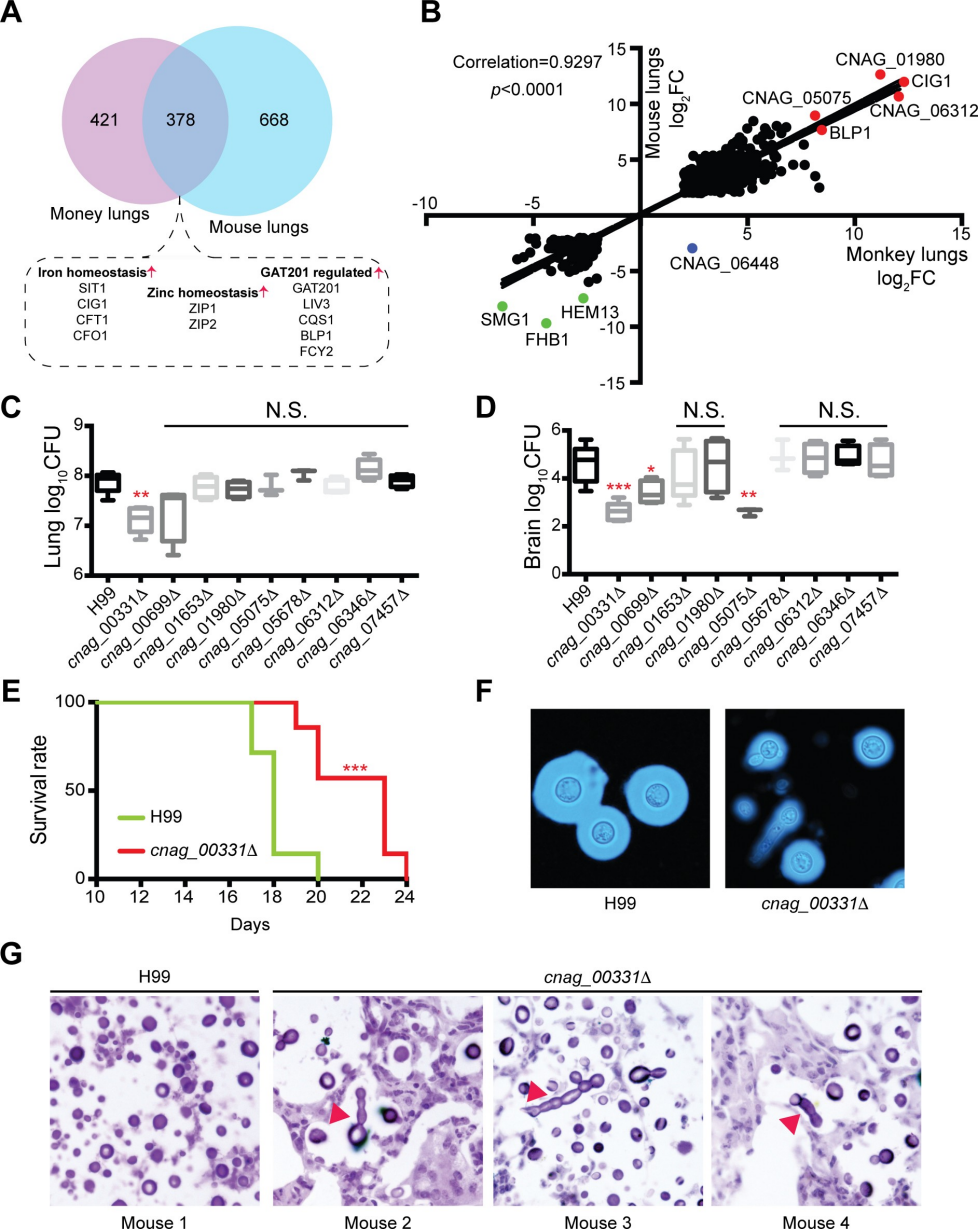
Identification of novel virulence factors in *Cryptococcus neoformans*. (A) Comparative transcriptome analysis of pulmonary *C*. *neoformans* in mice and monkeys. *Cryptococcus neoformans* genes from mouse and monkey lungs were analyzed using dual RNA sequencing, compared, and summarized using a Venn diagram. Important virulence modulators were identified and included genes involved in iron and zinc homeostasis and GAT201 regulation. (B) Correlation of *C*. *neoformans* genes that overlap between mouse and monkey lungs. The differentially expressed *C*. *neoformans* genes from mice and monkeys were plotted based on gene expression, and correlations and *p*-values were calculated. The most significantly induced and repressed genes are indicated, the former in red and the latter in green. (C) Lung CFU analysis of *C*. *neoformans* knockout mutants. Female C57BL/6 mice (*n* = 5) were intranasally infected with *C*. *neoformans* strains. Lung tissues were isolated 14 days postinfection, and CFUs were calculated. The Student *t* test was performed (**p < 0.01; N.S. indicates that significance was not found). (D) Brain CFU analysis of *C*. *neoformans* knockout mutants. Brain tissues from Fig 7C were isolated 14 days postinfection, and CFUs were calculated. The Student *t* test was performed (***p < 0.005, **p < 0.01, *p < 0.05; N.S. indicates that significance was not found). (E) Survival curve of animals infected with the *cnag_00331Δ* mutant. The experiment was performed as described in Fig 7C (*n* = 8). Animals were monitored for signs of infection. Statistical analysis was performed using the log-rank test (***p < 0.005). (F) Capsule production in *cnag_00331Δ* mutant cells. *Cryptococcus neoformans* strains were incubated in Dulbecco’s Modified Eagle supplemented with 10% FBS for 3 days. (G) Histopathology of infected mouse lung sections. Lung tissues from Fig 7C were fixed and stained with hematoxylin and eosin, then examined. Abnormal morphology in *C*. *neoformans* cells is indicated by red arrows.

Expression of 378 *C*. *neoformans* genes were altered in the lung tissues of both mice and monkeys during infections (Fig 7A). Of these genes, many that are reportedly involved in important biological processes allowing fungal adaptation in the host niche were differentially expressed. Genes involved in iron homeostasis (*SIT1*, *CIG1*, *CFT1*, and *CFO1*) and zinc homeostasis (*ZIP1* and *ZIP2*) are induced, suggesting that *C*. *neoformans* cells sense iron limitations in mice and monkeys. Genes expressions that are regulated by a transcription factor, Gat201, were also induced. They include *GAT201*, *LIV3*, *CQS1*, *BLP1*, and *FCY2*. Studies have extensively demonstrated the critical functions of genes in iron homeostasis, zinc acquisition, and Gat201 regulation in *C*. *neoformans* [6, 31–34]. Our data is consistent in that the host utilizes the immuno-defense mechanism against *C*. *neoformans* infection. Compared to the transcripts of *C*. *neoformans* cells in mice and monkeys, these 378 genes demonstrated good correlation (0.9297, *p* < 0.001) with the exception that *CNAG_06448* being expressed differentially between the two hosts (Fig 7B). The *BLP1* gene encoding the protein involved in cell surface architecture was abundantly expressed in both hosts, while *CIG1* involved in melanin formation and iron acquisition [32], was also highly induced upon infection in both species.

Significantly high expression levels indicated the importance of those genes in fungal pathogenicity. To explore these functions, 9 genes were selected for knockout studies (S4A Fig, S6 Table) and for virulence evaluation in mice. The *cnag_00331Δ* mutant demonstrated a strong reduction in fungal burden in the lung and the brain, while the *cnag_00699Δ* and *cnag_05075Δ* mutants showed lowered fungal burdens in the brain in comparison to the control (Fig 7C and 7D). When challenging mice with the wild-type or *cnag_00331Δ* mutant, a significant delay in death was also observed, indicating that *CNAG_00331* is an important virulence factor (Fig 7E).

Melanin formation and capsule production were also examined. While melanin formation of the *cnag_00331Δ* mutant did not differ from that of the wild-type strain (S4B Fig), a remarkable reduction in capsule size was observed (Fig 7F). Importantly, pseudohyphae-like cell morphology was also observed in the *cnag_00331Δ* mutant, even though only a small percentage of cells demonstrated this phenotype. Lung tissue cryosections were processed and stained to visualize the fungal morphology in the lung. Resembling that from the *in vitro* assay in Dulbecco’s Modified Eagle medium (DMEM), the *cnag_00331* mutant demonstrated an elongated and pseudohyphae-like structure during infection (Fig 7G). Collectively, these results revealed an important informative database for novel factors contributing to *C*. *neoformans* virulence.

## Discussion

*C*. *neoformans* is a leading cause of fungal meningoencephalitis in humans, yet efficient clinical treatment remains limited because it is greatly hampered by the lack of knowledge regarding interactions between fungal cells and the host at the infection axis [2, 35, 36]. Numerous studies have extensively focused on murine infection models to identify important virulence factors and host immune responses to infection. Through such efforts, various immune regulators have been identified to have important functions in *C*. *neoformans* infection progression and clearance. For example, IL-5 is significantly more prevalent following the exposure to *C*. *neoformans* cells [37]. Overexpressing the *IL-5* gene in a transgenic mouse model resulted in greater infiltration of pulmonary eosinophilia, and this model was more susceptible to *C*. *neoformans* infection [38]. Mice with overexpressed *IL-13* also showed reduced survival times and greater fungal proliferation in the lungs, indicating that IL-13 plays a role in triggering allergic inflammation during *C*. *neoformans* infections [39]. IL-17A protects the host from *C*. *neoformans* infection through leukocyte recruitment, activation, and gamma interferon production [20–22, 40]. While these findings are important, clinical investigations and *in vitro* studies employing cells of human origins revealed different sets of immune responses. *C*. *neoformans* cells increased the production of IL-8 and CXCL1 in human bronchial epithelial cells [41, 42]. CCL2, CXCL10, IL-10, and CCL3 are significantly elevated in patients with *C*. *neoformans* infection [20, 24, 42–44]. Notably, as with mice, IL-17A is induced in human patients with cryptococcal meningitis [20, 21]. Therefore, it is critical to realize the limit of murine experimental data in resembling clinical setting an urgent need to develop nonhuman primate models of cryptococcosis.

To mimic *C*. *neoformans* infections in humans, we developed a monkey infection model and we also carried our dual RNA-Seq to profile gene expressions of both the pathogen and the host. Comparisons of monkey with mouse greatly revealed the differences in host responses and demonstrated the feasibility of the nonhuman primate model towards understanding *C*. *neoformans* infections in humans. Our data revealed similar but distinct host responses to *C*. *neoformans* infections between mouse and monkey tissues. RNA-Seq analysis results were consistent with those of previously published findings. Specifically, IL-5 and IL-13, which have been shown to activate the defense response to *C*. *neoformans* infection [38, 39], were significantly induced in the mouse infection assay. From the monkey transcriptome, a significant number of immune regulators were also found to resemble those of the clinical setting but not of studies employing mice. Included are *CCL2*, *CCL3*, and *CXCL10*, which were statistically more abundant in humans infected with *C*. *neoformans* [24, 44]. In particular, our data showed that *CXCL10* was induced only in in infected monkey lungs, while it remains unchanged in the mouse tissues. IL-17A, with altered gene expression in both murine and human infections [20, 21], was more highly expressed in both our infection models. Taken together, these results demonstrate that the monkey infection model offers an infection protocol for more mimicking host responses in humans. Moreover, our data provide a set of immune regulators whose functions remain uninvestigated, and these results call for a throughout analysis of the functions of these genes in *C*. *neoformans* infections.

Intriguingly, comparative transcriptome analysis also revealed partially conserved defense mechanisms. Two hundred and twenty-five genes were differentially co-expressed in mice and monkeys, and they are important players in cytokine-cytokine receptor interactions, lysosomes, ferroptosis, and the cell cycle. While MMP12 serves no detectable function, OC-STAMP was shown to be important in defense against *C*. *neoformans* infections. Other studies demonstrated that OC-STAMP-deficient mice exhibited a lack of cell-cell fusion in macrophage-lineage multinuclear cells [25, 26] and the expression of *OC-STAMP* was induced by RANKL [25]. Our data showed that the gene expressions of both *RANKL* and *OC-STAMP* were dramatically induced upon *C*. *neoformans* infection in the lungs of both models. Furthermore, we showed that Cas9-mediated *CO-STAMP* knockout mice exhibited a phenotype resistance to *C*. *neoformans* infection. We speculated that resistance to fungal infections in *OC-STAMP*^*-/-*^ mice is most likely the result of inhibiting cell-cell fusion in macrophage lineage cells and increasing the macrophage-to-fungal-cell ratios [26]. The macrophages represent the majority of immune cells in a host defending against *C*. *neoformans*.[45].

Depleting of the insulin-producing cells rendered in mice more susceptible to *C*. *neoformans* infection. In fact, people with diabetes are more sensitive to viral and *Mycobacterium tuberculosis* infections [46]. In diabetic mice, it has recently been shown that IL-17 production is enhanced, causing the activation of microbiome pathogenicity [47]. Furthermore, a recent clinical investigation revealed that type 2 diabetic mellitus contributed to the incidence of *C*. *neoformans* infections in China [48]. We showed that *C*. *neoformans* significantly modulates the expression of key players of the insulin-signaling process in tested animals. Damaging insulin-producing cells caused higher fungal burden in the lung tissue and shorter survival, whereas modulating the host sugar level had no impact on the fungal resistance. Previous studies demonstrated that diabetic mice dampen a wide range of immune responses. These include reduced phagocytosis and chemokine production, affected macrophage and neutrophil recruitment, delayed T cell trafficking to lung and others (reviewed in [49]). Therefore, these data underscored the potential susceptibility of diabetic individuals to *C*. *neoformans* infection.

Finally, in response to the different host environment, 378 genes were differentially expressed in mouse and monkey lung tissues where *C*. *neoformans* dampens glycolysis and TCA cycles but induces ribosome biogenesis, iron and zinc homeostasis, and the GAT201 regulation process. Our research efforts also resulted in the identification of novel regulators of fungal virulence, including *CNAG_00331*. Disrupting *CNAG_00331* resulted in the formation of cells with pseudohyphae-like structure. Since *C*. *neoformans* morphological transition also plays a vital role in proliferation and thus fitness during pulmonary infection [11]. Our characterization of *CNAG_00331* as a novel virulence factor were consistent with those notion.

In conclusion, this research represented a significant advancement in infection model development for *C*. *neoformans* studies and demonstrated that dual RNA-Seq analysis can be used efficiently to profile expressions of both the pathogen and the host during the disease interaction. Our study also provided critical data aiding in the elucidation of virulence mechanism that facilitates novel anti-fungal therapeutic targets.

## Material and methods

### Strains and media

*C*. *neoformans* strains (S6 Table) were routinely grown in YPD medium (1% yeast extract, 2% peptone, 2% glucose) at 30°C. The wild-type strain H99 was used to generate nine knockout mutants. Nine genes including *CNAG_00331*, *CNAG_00699*, *CNAG_01653*, *CNAG_01980*, *CNAG_05075*, *CNAG_05678*, *CNAG_06312*, *CNAG_06346*, and *CNAG_07457*, were deleted by overlapping PCR method [50]. Integration cassettes were constructed using overlap PCR. Upstream and downstream sequences of target genes were amplified using primers (S7 Table), and the resulting PCR fragments were gel purified and joined with the neomycin resistance marker via PCR. A helium gas biolistic device was used for *C*. *neoformans* transformation, as described previously [50, 51]. Transformants were grown and selected from YPD agar (1% yeast extract, 2% peptone, 2% glucose, 2% agar) supplemented with 200 μg/mL G418.

### Ethics statement

Infection experiments in mice and monkeys were reviewed and ethically approved by the Research Ethics Committees of the College of Life and Health Sciences of Northeastern University (Approval no. 16099M) and Wincon TheraCells Biotechnologies Co., Ltd. (Approval no. WD-20150701-a). All animal experiments were carried out in accordance with the regulations in the Guide for the Care and Use of Laboratory Animals issued by the Ministry of Science and Technology of the People's Republic of China. Cynomolgus monkeys (*Macaca fascicularis*) care and use by Wincon TheraCells Biotechnologies Co., Ltd. with an alternating 12 h light-dark cycle and uninterrupted water supply. Food was available twice a day and supplemented with fresh fruits. Monkeys were anesthetized with ketamine, deep anesthesia was induced by injection with an overdose of pentobarbital, and sacrificed. Mice housed in the College of Life and Health Sciences of Northeastern University were cared with an alternating 12 h light-dark cycle and unlimited food and water supply. Mice were anesthetized by ketamine and sacrificed by exposure to CO_2_ for 5 min.

### Animal infections

Mice and cynomolgus monkeys were infected with *C*. *neoformans* H99 strain. Six eight-week-old female C57BL/6 mice (purchased from Changsheng Biotech, China) were used [13]. Mice were anesthetized prior to infection intranasally with 10^5^ fungal cells in 50 μL PBS. Mice were monitored twice daily for signs of infection and humanely killed when endpoints were reached. Mice were sacrificed 7 days postinfection and lung and brain tissues were collected. Tissues were isolated and resuspended in RNAlater (Thermofisher) and RNA samples were isolated for RNA-Seq. Tissues were also collected from control mice infected with PBS. Fungal burdens were determined using lung tissues 7 and 14 days postinfection. Tissues were weighed, homogenized in PBS, and diluted prior to plating onto YPD agar medium at 30°C for 3 days.

Cynomolgus monkey infection care was performed by Wincon TheraCells Biotechnologies Co., Ltd., that was endorsed by the Association for Assessment and Accreditation of Laboratory Animal Care (No. W00099). Six female cynomolgus monkeys were purchased from Grandforest Co., Guangxi, China and divided into two groups: controls (aged 5.55 ± 1.39 years and weight 3.43 ± 0.21 kg) and the infection group (aged 7.24 ± 1.01 years and weight 4.13 ± 1.47 kg). Monkeys were anesthetized via intraperitoneal injections of ketamine (10 mg/kg). H99 cells were washed three times with PBS buffer, and counted. Monkeys were infected via intratracheal injection with 10^8^ cells/kg *C*. *neoformans* H99 cells. Controls were infected using PBS. Animals were monitored daily for body temperature signs of stress associated with infections including cough, fever, and changes in eating behavior and mobility. Seven days following infection, monkeys were sacrificed and lung tissues collected. Tissues were either fragmented into 0.5 cm × 0.5 cm × 0.5 cm cubes prior to suspending in either an RNAlater solution (Ambion) or paraformaldehyde solutions.

### Histopathology and colony forming units (CFU) assessment

Lung and brain tissues were collected from mice and monkeys [9, 13]. To perform histopathology analyses, tissue samples were fixed with paraformaldehyde, frozen, and processed using a cryostat microtome (CM1850; Leica). Tissue sections of 10 μm in thickness were stained with mucicarmine or hematoxylin/eosin. For CFUs determination, tissues were weighed, homogenized, diluted, and plated onto YPD agar at 30°C for 3 days. Cell colonies were counted and calculated.

### RNA sequencing library preparation and transcriptome analysis

Total RNA was isolated using Trizol (Invitrogen Life Technologies). RNA concentration, quantitation, and integrity assessment were done using a NanoDrop 8000 spectrophotometer (Thermo Scientific). Sequencing libraries were generated using the TruSeq RNA Sample Preparation Kit (Illumina, San Diego, CA, USA) based on the standard protocol. Briefly, mRNA was purified from total RNA using poly-T oligo-attached magnetic beads. First strand cDNA was synthesized using random oligonucleotides and SuperScript II (Thermo Scientific). Second strand cDNA was synthesized using DNA Polymerase I and RNase H. After adenylation of the 3′ ends of the DNA fragments, Illumina PE adapter oligonucleotides were ligated. Ligated products were selectively enriched using Illumina PCR Primer Cocktail in a 15-cycle PCR reaction. Products were the purified (AMPure XP system) and quantified using the Agilent high-sensitivity DNA assay and an Agilent 2100 Bioanalyzer System (Agilent Technologies). Libraries were then sequenced on a Nextseq 500 platform (Illumina) with paired-end 2×150-bp reads. Both RNA libraries construction and sequencing were performed by Shanghai Personal Biotechnology Cp. Ltd. (Shanghai, China).

Raw RNA-Seq data were preprocessed, assembled, and then filtered using standard QC criteria. The corresponding sequenced reads were mapped to the mouse, monkey, and *C*. *neoformans* genomes in NCBI (https://www.ncbi.nlm.nih.gov/genome/) using Bowtie/Tophat (http://ccb.jhu.edu/software/tophat/index.shtml). Fold changes were calculated using DESeq2 (http://bioconductor.org/packages/release/bioc/html/DESeq2.html). Differential expression deemed significant if the changes are at least 2-fold and *p-adj* is < 0.05.

### Enzyme-Link Immunosorbent Assay (ELISA)

ELISAs of CCL2, CCL22, CCL2 and CCL7 (Thermo Fisher Scientific) were performed using lung tissues of *C*. *neoformans* infected mice and monkeys, and tissues of PBS-infected were used as controls. Briefly, tissues were homogenized and protein samples were isolated and quantified using ELISA kits, according to the manufacture’s manuals.

### CRISPR-Cas9 knockout mice

*OC-STAMP*^*-/-*^ and *MMP12*^*-/-*^ knockout mice were produced by Beijing View Solid Biotechnology, China. Briefly, the pCAG-T7-SaCas9 plasmid linearized by *Avr*II restriction was used as an *in vitro* transcriptional template. After purification, saCas9 mRNA was transcribed with the mMESSAGE mMACHINE™ T7 ULTRA Transcription Kit (Life Technology). Target gRNA templates were amplified based on the gRNA scaffold using T7 promoter sequence-conjugated primers (S7 Table) and then transcribed using a fast *in vitro* transcription T7 kit (cat. no. VK010, Beijing View Solid Biotechnology, China) and frozen at -80°C. Zygotes of C57BL/6 mice were injected with saCas9 mRNA and target gRNA in M2 media (Millipore) using a FemtoJet micromanipulator (Eppendorf, Germany). Following microinjection, zygotes were transferred to pseudo pregnant female mice. All mice were maintained in a specific pathogen-free facility. The tail-derived DNA from 2-week-old newborn mice was genotyped by sequencing the PCR products amplified by primers (S7 Table). Mutant mice were mated with wild-type C57BL/6 mice to obtain heterozygous knockout mice.

### Streptozotocin treatment and glucose measurements

Blood glucose levels in infected and non-infected mice were monitored daily. Blood samples were drawn by pinpricking the tail, and blood glucose was measured using an Accusure 580 (Yuwell, China). Glucose levels in the cerebrospinal fluid (CSF) were measured using collected CSF and levels in the brain were measured using homogenized brain tissues.

Correlations between insulin secretion and *C*. *neoformans* infection were evaluated using an induced diabetic mouse model was described previously [52]. Six- to eight-week-old female C57BL/6 mice were treated with a streptozotocin (STZ) sodium citrate solution (150 mg/kg) after fasting for 4 hours. Treated mice were supplied with regular food and sucrose drinking water (10%) for 48 hours. The latter was later replaced with unmodified water. Mice continuously demonstrating high blood glucose levels (over 300 mg/dL) were considered to demonstrate signs of diabetes and were used for further experiments.

The resistance of high sugar- or fat-diet fed mice to *C*. *neoformans* infection was assayed. Briefly, mice were divided into three groups: control group (normal chow and water, n = 7), high-sugar group (normal chow and 30% w/v of sucrose solution, n = 7), and high-fat group (high-fat chow and normal water, n = 7). Body weights and blood sugar levels were monitored for 7 days. Mice continuously demonstrating high blood sugar levels were used for CFU analysis.

### GO analysis and regulation network constructions

Gene ontology and KEGG analyses were performed using R 3.4.3, clusterprofiler 3.6.0, Annotation Hub (2.12.1), and org.Mm.eg.db (3.60) packages [53]. The databases for cynomolgus monkeys and mice were downloaded from Annotation Hub and org.Mm.eg.db, respectively. *C*. *neoformans* gene ontology data were downloaded from Annotation Hub. The EnrichGO function was used for analysis, and KOBAS 3.0 was employed to perform KEGG analyses for *C*. *neoformans*.

### Capsule production and microscopic observation

Capsular structures were induced by incubating H99 cells in DMEM supplemented with 10% fetal calf serum (FCS) at 37°C and under 5% CO_2_ for 3 days. The capsule was visualized by Indian ink staining [9].

Animal lung tissues were processed and stained with mucicarmine or hematoxylin/eosin staining. Tissues were visualized under a Leica DM4000B LED microscope with a 20x objective (Leica). The capsule was observed and photographed using a Nikon Eclipse Ni-E microscope with a 100x oil objective (Nikon).

## Supporting information

S1 Fig Reproducibility of RNA-Seq results. (A) Raw read counts for mouse and monkey tissues were plotted. The Pearson correlation coefficient was calculated for each comparison. (B) Raw read counts for *C*. *neoformans* were plotted. The Pearson correlation coefficient was calculated for each comparison.
(TIF)

S2 Fig Alterations in gene-encoding immune signals in mouse and monkey lung tissues. Changes in gene expression of chemokines, cytokines, the TNF family, and the TGF family in response to *C*. *neoformans*, as indicated by arrows.
(TIF)

S3 Fig Generating *OC-STAMP^-/-^* knockout mouse lines. (A)Scheme used to generate Cas9-mediated *OC-STAMP*^*-/-*^ knockout mice. Exon 2 was selected for recognizing gRNA-Cas9. (B) Confirmation of *OC-STAMP*^*-/-*^ knockout mice. In *OC-STAMP*^*-/-*^_1 knockout mice, a 202-bp genomic DNA fragment of exon 2 was deleted. The PCR products from wild-type mice generated a 476-bp band, and homozygous knockout mice generated a 274-bp product. In *OC-STAMP*^*-/-*^_2 knockout mice, a 17-bp genomic DNA fragment of exon 2 and a 38-bp fragment of intron 1–2 were deleted. The PCR products from wild-type mice generated a 200-bp band, and those from homozygous knockout mice generated a 145-bp product. In *OC-STAMP*^*-/-*^_3 knockout mice, a 7-bp genomic DNA fragment of exon 2 was deleted. The PCR products from wild-type and knockout mice generated similar sized products. (C) In wild-type and the *OC-STAMP*^*-/-*^_3 line, DNA sequencing analyses were performed to confirm the deletion.
(TIF)

S4 Fig Capsule formation and melanin production of *Cryptococcus neoformans* mutants. (A) Capsule formation of *Cryptococcus neoformans* mutants. Strains of *C*. *neoformans* were grown in Dulbecco’s Modified Eagle medium (with 10% FBS) for 2 days. Fungal cells were strained with Indian ink and microscopically photographed. Capsule structure thicknesses were quantified. (B) Melanin production. The wildtype H99 strain and *cnag_00331Δ* strain were spotted onto L-DOPA agar plates. Photos were taken after 3 days of incubation at 37°C.
(TIF)

S1 Table Tissue RNA-Seq analyses of mouse and monkey in response to *C. neoformans* infection. (XLSX)

S2 Table KEGG analysis of genes differentially expressed in *C. neoformans* infected lungs. (XLSX)

S3 Table Differentially expressed *C. neoformans* genes in the mouse and monkey lungs. (XLSX)

S4 Table KEGG and GO analyses of *C. neoformans* transcriptome in mouse lung tissues. (XLSX)

S5 Table KEGG and GO analyses of *C. neoformans* transcriptome in monkey lung tissues. (XLSX)

S6 Table Strains used in this study. (XLSX)

S7 Table Primers used in this study. (XLSX)
