# Supplementary figures and images for: Unveil the transcriptional landscape at the Cryptococcus-host axis in mice and nonhuman primates
Source: PLoS Negl Trop Dis. 2019 Jul 22;13(7):e0007566. doi: 10.1371/journal.pntd.0007566 (PMC6675133; doi:10.1371/journal.pntd.0007566)

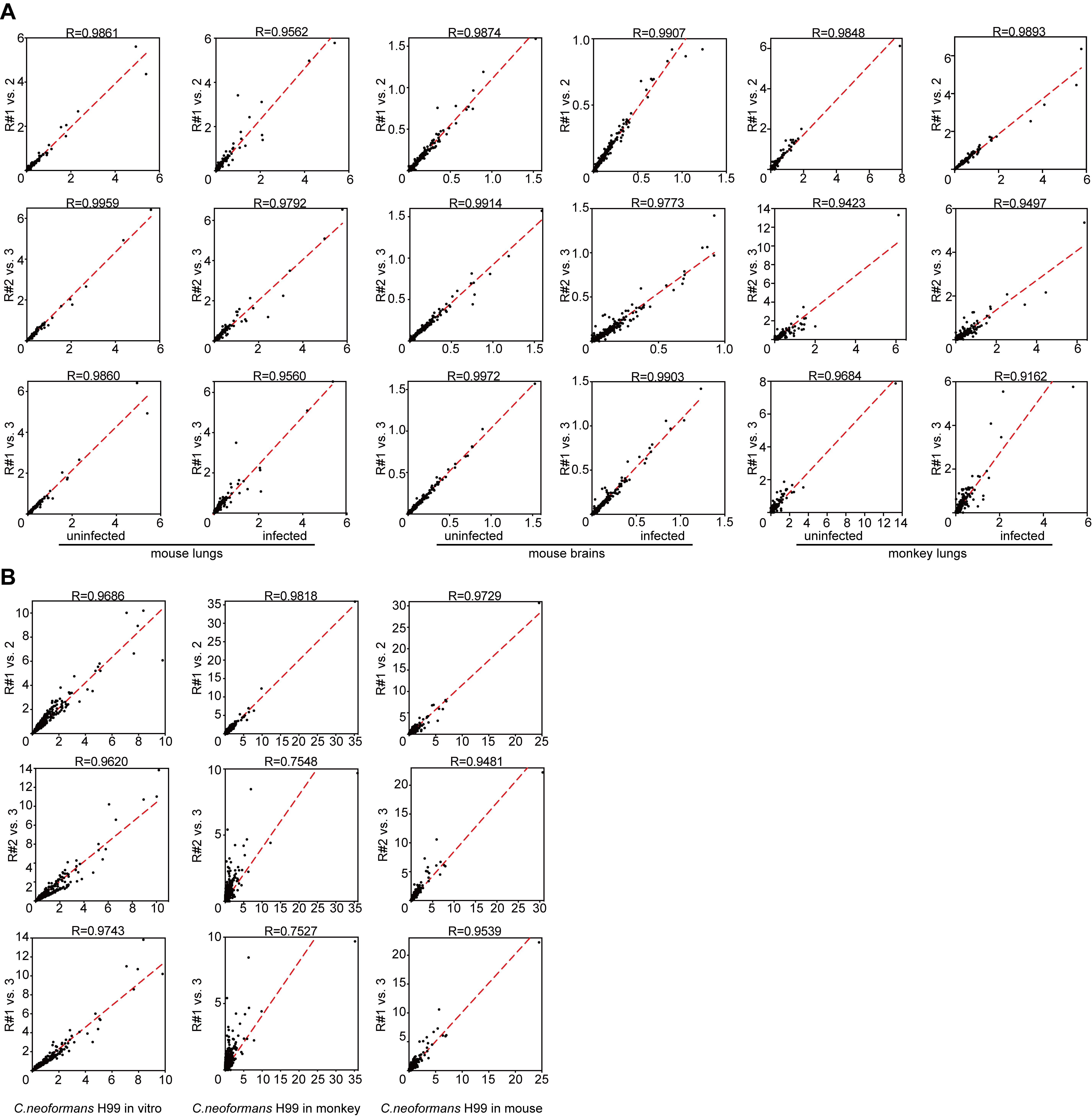

Supplement: S1 Fig — (A) Raw read counts for mouse and monkey tissues were plotted. The Pearson correlation coefficient was calculated for each comparison. (B) Raw read counts for C. neoformans were plotted. The Pearson correlation coefficient was calculated for each comparison. (TIF) [file pntd.0007566.s001.tif]

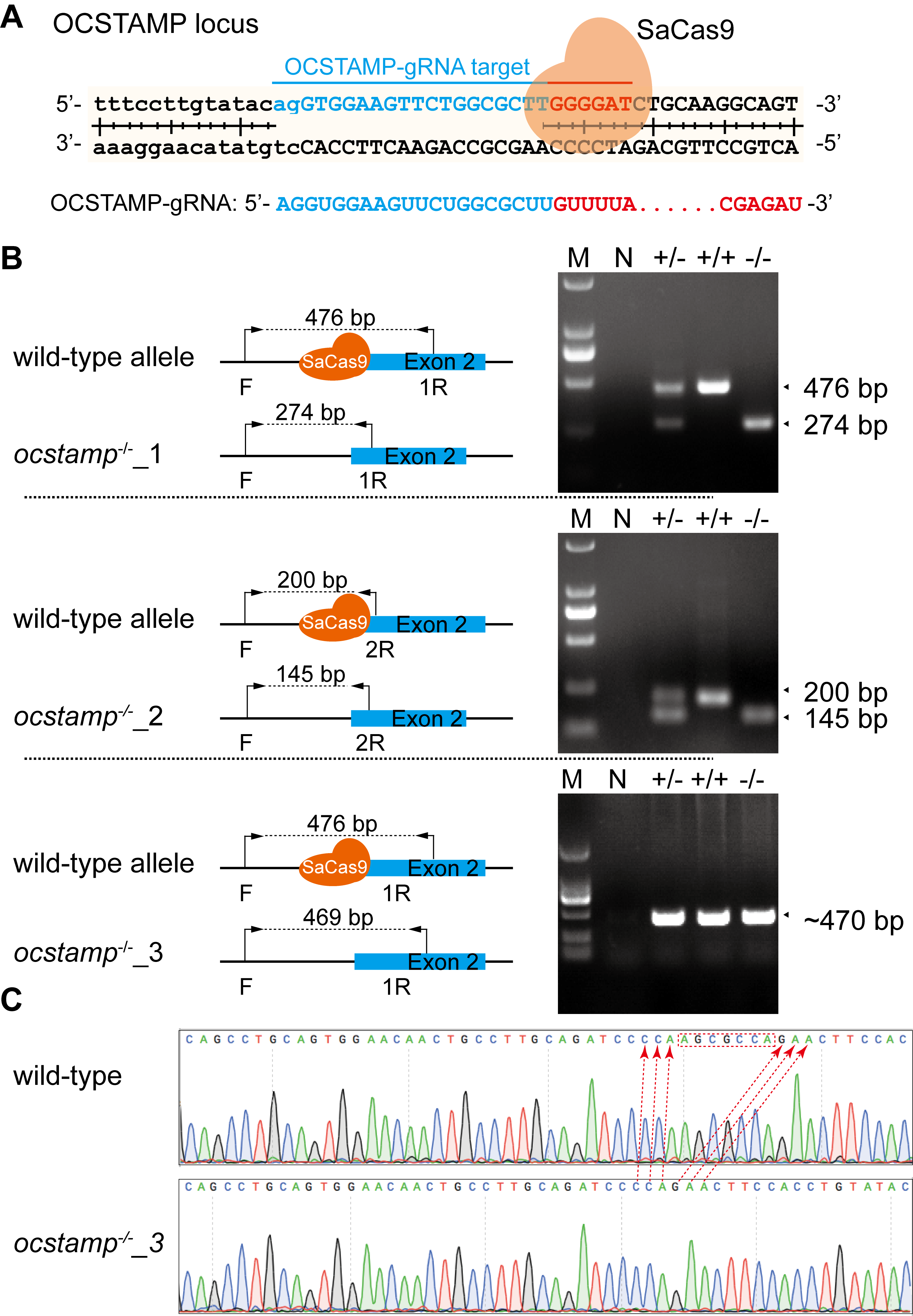

Supplement: S3 Fig — (A)Scheme used to generate Cas9-mediated OC-STAMP-/- knockout mice. Exon 2 was selected for recognizing gRNA-Cas9. (B) Confirmation of OC-STAMP-/- knockout mice. In OC-STAMP-/-_1 knockout mice, a 202-bp genomic DNA fragment of exon 2 was deleted. The PCR products from wild-type mice generated a 476-bp band, and homozygous knockout mice generated a 274-bp product. In OC-STAMP-/-_2 knockout mice, a 17-bp genomic DNA fragment of exon 2 and a 38-bp fragment of intron 1–2 were deleted. The PCR products from wild-type mice generated a 200-bp band, and those from homozygous knockout mice generated a 145-bp product. In OC-STAMP-/-_3 knockout mice, a 7-bp genomic DNA fragment of exon 2 was deleted. The PCR products from wild-type and knockout mice generated similar sized products. (C) In wild-type and the OC-STAMP-/-_3 line, DNA sequencing analyses were performed to confirm the deletion. (TIF) [file pntd.0007566.s003.tif]

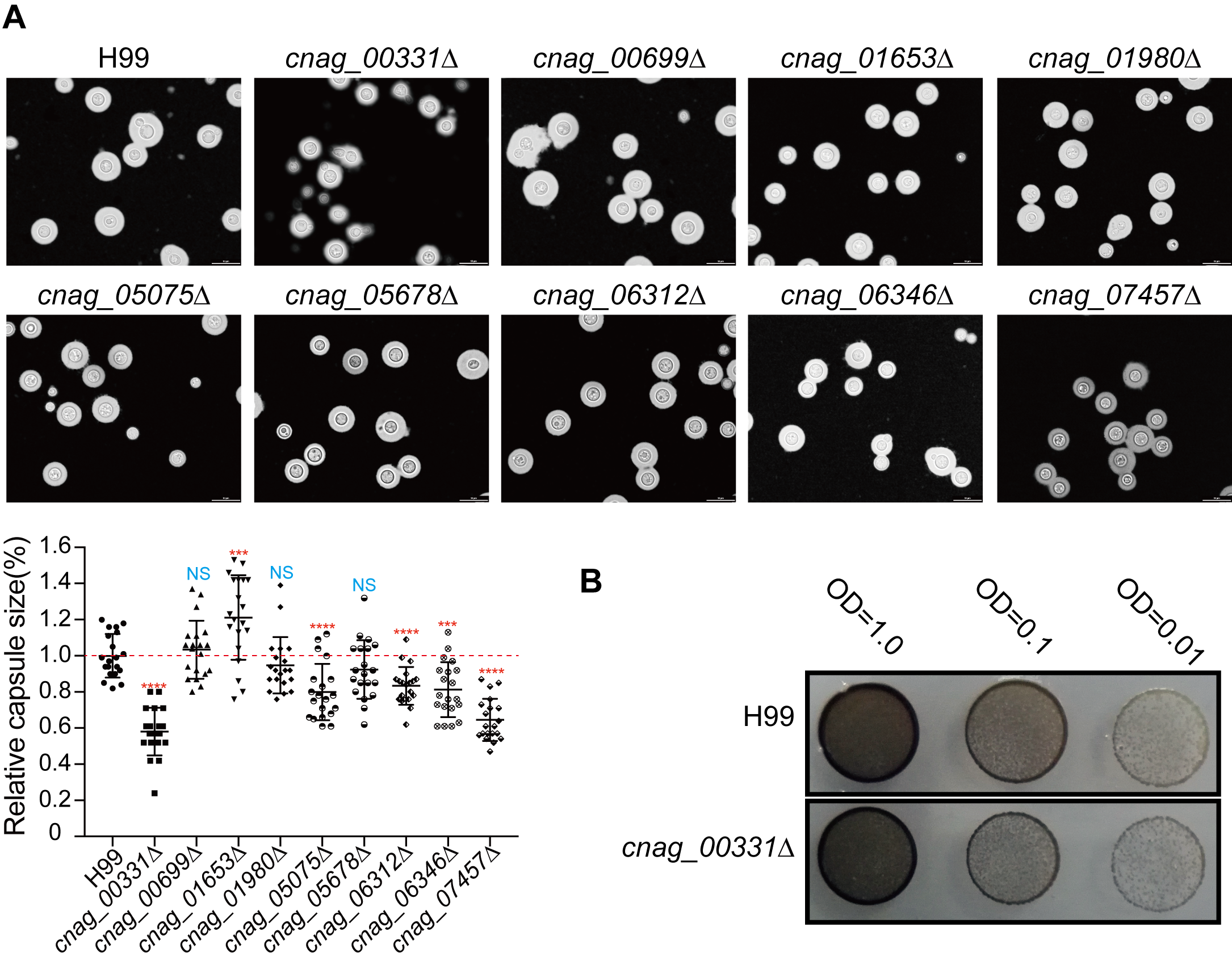

Supplement: S4 Fig — (A) Capsule formation of Cryptococcus neoformans mutants. Strains of C. neoformans were grown in Dulbecco’s Modified Eagle medium (with 10% FBS) for 2 days. Fungal cells were strained with Indian ink and microscopically photographed. Capsule structure thicknesses were quantified. (B) Melanin production. The wildtype H99 strain and cnag_00331Δ strain were spotted onto L-DOPA agar plates. Photos were taken after 3 days of incubation at 37°C. (TIF) [file pntd.0007566.s004.tif]
